# Current practice and effects of intravenous anticoagulant therapy in hospitalized acute heart failure patients with sinus rhythm

**DOI:** 10.1038/s41598-020-79700-5

**Published:** 2021-01-13

**Authors:** Hiroki Nakano, Yasuhiro Hamatani, Toshiyuki Nagai, Michikazu Nakai, Kunihiro Nishimura, Yoko Sumita, Hisao Ogawa, Toshihisa Anzai

**Affiliations:** 1 Department of Cardiovascular Medicine, National Cerebral and Cardiovascular Center, Osaka, Japan; 2 Department of Cardiology, Tokyo Medical University, Tokyo, Japan; 3 Department of Cardiology, National Hospital Organization Kyoto Medical Center, Kyoto, Japan; 4 Department of Cardiovascular Medicine, Faculty of Medicine and Graduate School of Medicine, Hokkaido University, Kita 5, Nishi 8, Kita-ku, Sapporo, Japan; 5 Center for Cerebral and Cardiovascular Disease Information, National Cerebral and Cardiovascular Center, Osaka, Japan; 6 Department of Preventive Medicine, National Cerebral and Cardiovascular Center, Osaka, Japan

**Keywords:** Cardiology, Cardiovascular diseases

## Abstract

Although the risk of thromboembolism is increased in heart failure (HF) patients irrespective of atrial fibrillation (AF), especially during the acute decompensated phase, the effects of intravenous anticoagulants for these patients remain unclear. We sought to investigate the current practice and effects of intravenous anticoagulant therapy in acute HF (AHF) patients with sinus rhythm. We analyzed a nationwide prospective cohort from April 2012 to March 2016. We extracted 309,015 AHF adult patients. After application of the exclusion criteria, we divided the 92,573 study population into non-heparin [n = 70,621 (76.3%)] and heparin [n = 21,952 (23.7%)] groups according to the use of intravenous heparin for the first 2 consecutive days after admission. Multivariable logistic regression analyses demonstrated that heparin administration was not associated with in-hospital mortality (OR 0.97, 95% CI 0.91–1.03) and intracranial hemorrhage (OR 1.18, 95% CI 0.78–1.77), while heparin administration was significantly associated with increased incidence of ischemic stroke (OR 1.49, 95% CI 1.29–1.72) and venous thromboembolism (OR 1.62, 95% CI 1.14–2.30). In conclusion, intravenous heparin administration was not associated with favorable in-hospital outcomes in AHF patients with sinus rhythm. Routine additive use of intravenous heparin to initial treatment might not be recommended in AHF patients.

## Introduction

Heart failure (HF) is a major growing public health problem worldwide in the aging society. Despite advances in the management of HF, the morbidity and mortality of HF patients are still high^1^. Thromboembolism such as ischemic stroke and venous thromboembolism (VTE) is a devastating morbidity of HF and contributes to a poor prognosis in HF patients^2,3^. In fact, atrial fibrillation (AF) commonly coexists with HF and increases the risk of thromboembolism. Nevertheless, HF per se is an important risk factor for thromboembolism. Although the efficacy of oral anticoagulant therapy for prevention of thromboembolism in patients with AF has been proven^4^, there are few reports demonstrating the overall beneficial effects of anticoagulant therapy in HF patients without AF^5^. Notably, HF increases the risk of thromboembolism through the fulfilment of Virchow’s triad for thrombogenesis^6^. In patients with acute HF (AHF), the coagulation system and endothelial function are more severely impaired than in those with chronic HF. Moreover, higher intracardiac pressure, reduced ventricular contraction, and hemoconcentration due to diuretics further predispose AHF patients to higher risk of thromboembolism. Previous reports showed that the risk of thromboembolism was markedly high in the short-term after the onset of AHF^7–11^. Thus, current HF guidelines recommend routine thromboembolism prophylaxis with heparin or other anticoagulants in patients with AHF unless contraindicated^12^. However, despite this recommendation, thromboembolism prophylaxis may be underutilized in AHF patients due to the paucity of evidence verifying the efficacy and safety of intravenous anticoagulant therapy especially in AHF patients with sinus rhythm. Accordingly, the aims of this study were to investigate the current practice and effects of additive use of intravenous anticoagulant therapy to initial treatment on outcomes including in-hospital death, thromboembolism and bleeding in hospitalized AHF patients with sinus rhythm, using a nationwide claim database.

## Methods

### Data source

All data were extracted from the Japanese Registry Of All cardiac and vascular Diseases-Diagnosis Procedure Combination (JROAD-DPC). Briefly, the JROAD-DPC is a multicenter, observational, prospective cohort that involves the collection of administrative data from nearly all teaching hospitals with cardiovascular beds. Teaching hospitals participate in this project to meet the Japanese Circulation Society (JCS) cardiology training requirement for physicians who wish to be JCS board-certified cardiologists and take the JCS board test, as described previously^1^. This study was performed in the principles of the Declaration of Helsinki. Since the present research involves an observational study not using human biological specimens, it was waived the requirement for individual informed consent by using the “opt-out” principle according to the ethical guidelines for epidemiological research issued by the Ministry of Health, Labour and Welfare, Japan. This study has been approved by the Institutional Review Board of the National Cerebral and Cardiovascular Center (M30-030).

### Study population

**Figure 1:**
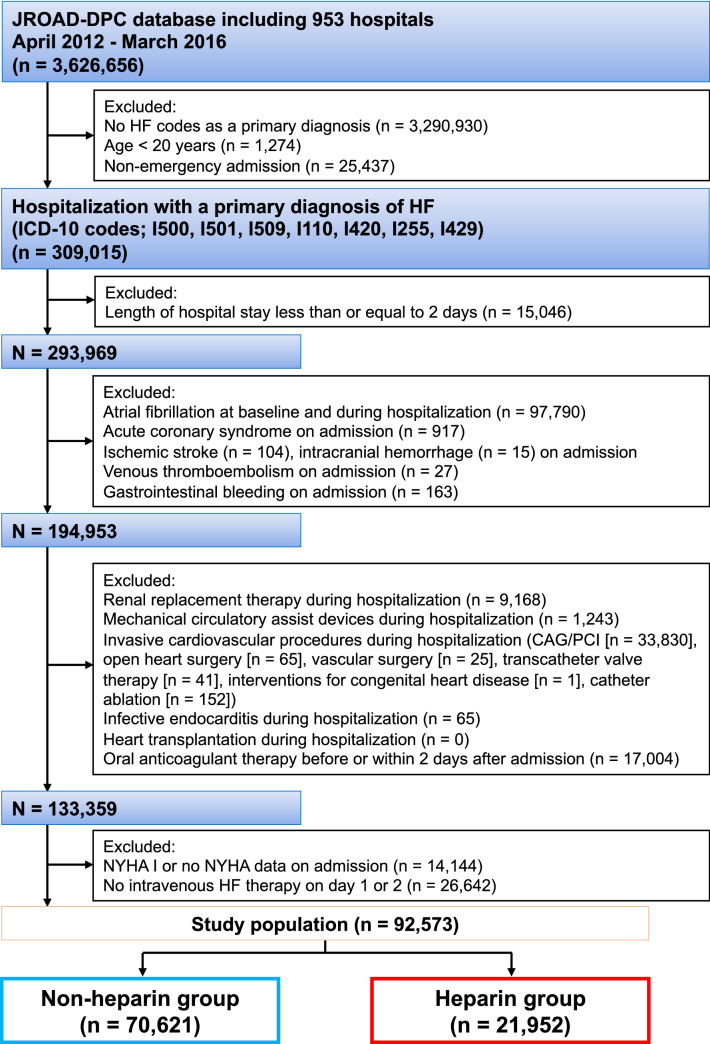
Flow diagram of this study. *CAG* coronary angiography, *HF* heart failure, *ICD-10* International Classification of Diseases 10th revision, *JROAD-DPC* Japanese Registry of All cardiac and vascular Diseases-Diagnosis Procedure Combination, *NYHA* New York Heart Association, *PCI* percutaneous coronary intervention.

We extracted 309,015 AHF patients aged 20 years or older who required emergent hospitalization between April 2012 and March 2016 according to the International Classification of Diseases 10th revision (ICD-10) codes I50.0, I50.1, I50.9, I11.0, I42.0, I25.5, and I42.9. Exclusion criteria are shown in Fig. 1 as follows: (1) age < 20 years, (2) non-emergency admission, (3) length of hospital stay ≤ 2 days, (4) AF at baseline and during hospitalization, (5) acute coronary syndrome, stroke (ischemic stroke and intracranial hemorrhage [ICH]), VTE or gastrointestinal bleeding on admission, (6) renal replacement therapy, mechanical circulatory assist devices and invasive cardiovascular procedures (coronary angiography/percutaneous coronary intervention, open heart surgery, vascular surgery, transcatheter valve therapy, interventions for congenital heart disease and catheter ablation) during hospitalization, (7) infective endocarditis during hospitalization, (8) heart transplantation during hospitalization, (9) oral anticoagulant therapy before or within 2 days after admission, (10) New York Heart Association (NYHA) functional class I or no NYHA data on admission, (11) no intravenous HF therapy (diuretics, vasodilators, inotropes or vasopressors) on day 1 or 2 after admission. ICD-10 and procedure codes related to these exclusion criteria are shown in Supplementary Appendix. Finally, a total of 92,573 AHF patients with sinus rhythm were included in this study. Then, we divided the study population into non-heparin and heparin groups according to the use of more than 10,000 units of heparin for the first 2 consecutive days after admission.

### Clinical outcomes

The primary outcome was in-hospital death. Secondary efficacy outcomes were in-hospital ischemic stroke and in-hospital VTE, and secondary safety outcomes were in-hospital ICH and composite bleeding (gastrointestinal bleeding and ICH). ICD-10 and procedure codes related to these outcomes are shown in Supplementary Appendix.

### Statistical analysis

Continuous variables are presented as mean ± standard deviation when normally distributed, and as median and interquartile range when non-normally distributed. Comparisons of differences among groups were performed by unpaired Student’s *t*-test or Mann–Whitney U test for continuous variables and chi-squared test for dichotomous variables as appropriate. We constructed multivariable logistic regression models to evaluate the association between heparin use and outcomes (in-hospital death, in-hospital ischemic stroke, in-hospital VTE, in-hospital ICH and in-hospital composite bleeding). Ten case-mix variables (age, sex, admission route, NYHA class, respiratory support, ischemic heart disease, hypertension, life-threatening arrhythmia, chronic kidney disease and shock), which were validated as important predictors of in-hospital death in patients with AHF from a Japanese DPC claim cohort (c-index: 0.80, 95% confidence interval [CI] 0.78–0.82), and the use of intravenous vasopressors or inotropes were adopted for adjustment of the primary outcome^13,14^. To evaluate the influence of heparin on secondary outcomes, we constructed the following two models: model 1, adjusted by specific covariates of each outcome; and model 2, model 1 with covariates that were used for adjustment of in-hospital death. Based on previous reports, variables of CHA_2_DS_2_VASc score as determinants of ischemic stroke, cardiovascular risk factors (hypertension, diabetes mellitus, hyperlipidemia and body mass index) as determinants of VTE, and variables of modified HASBLED score (hypertension, chronic kidney disease, liver disease, history of stroke, history of bleeding, age ≥ 65 years, therapy with either nonsteroidal anti-inflammatory drugs or anti-platelet agents, and alcoholism) as determinants of bleeding (ICH and composite bleeding) were used for adjustment of secondary outcomes in model 1^4,15–17^. All tests were two tailed, and a value of *P* < 0.05 was considered statistically significant. All analyses were performed with Stata MP64 version 15 (StataCorp, College Station, TX, USA).

## Results

### Baseline characteristics

Median age and length of hospital stay were 81 years and 23 (12—27) days, respectively. Among the 92,573 studied patients, 21,952 (24%) patients received intravenous heparin during the first 2 consecutive days of HF hospitalization (Fig. 1). Baseline characteristics are shown in Table 1. Patients treated with heparin were younger and predominantly male sex, and more frequently exhibited emergency admission with an ambulance, NYHA class IV, comorbidities such as ischemic heart disease, atherosclerotic risk factors and vascular disease than those without. Patients in the heparin group more frequently received intravenous inotropes, intravenous vasopressors and respiratory support. CHA_2_DS_2_VASc score and modified HASBLED score were higher in the heparin group than in the non-heparin group.

**Table 1:** Baseline characteristics.

Variable	Overall (*n* = 92,573)	Non-heparin group (*n* = 70,621)	Heparin group (*n* = 21,952)	*p*-value
Age, years	81.3 ± 11.7	81.7 ± 11.5	80.3 ± 12.0	< 0.001
**Age group, years, n (%)**				< 0.001
20−64	8299 (9.0)	5953 (8.4)	2346 (10.7)	
65−74	11,643 (12.6)	8629 (12.2)	3014 (13.7)	
≥ 75	72,631 (78.5)	56,039 (79.4)	16,592 (75.6)	
Female sex, n (%)	47,936 (51.8)	37,421 (53.0)	10,515 (47.9)	< 0.001
**Admission route, n (%)**				< 0.001
Scheduled outpatient clinic	14,135 (15.3)	12,109 (17.2)	2026 (9.2)	
Emergency department without an ambulance	38,198 (41.3)	29,939 (42.4)	8259 (37.6)	
Emergency department with an ambulance	40,222 (43.5)	28,562 (40.5)	11,660 (53.1)	
Body mass index, kg/m^2^	21.9 ± 5.9	21.8 ± 6.1	22.1 ± 5.3	< 0.001
**NYHA class, n (%)**				< 0.001
II	21,820 (23.6)	17,036 (24.1)	4784 (21.8)	
III	33,223 (35.9)	25,813 (36.6)	7410 (33.8)	
IV	37,530 (40.5)	27,772 (39.3)	9758 (44.6)	
Length of hospital stay, days	23 (12 – 27)	23 (11 – 27)	22 (12 – 27)	< 0.001
**Comorbidities, n (%)**				
Ischemic heart disease	24,369 (26.3)	17,826 (25.2)	6543 (29.8)	< 0.001
Dyslipidemia	16,952 (18.3)	12,101 (17.1)	4851 (22.1)	< 0.001
Diabetes mellitus	26,485 (28.6)	19,796 (28.0)	6689 (30.5)	< 0.001
Hypertension	49,885 (53.9)	37,344 (52.9)	12,541 (57.1)	< 0.001
Stroke	1850 (2.0)	1400 (2.0)	450 (2.1)	0.53
Venous thromboembolism	625 (0.7)	419 (0.6)	206 (0.9)	< 0.001
Vascular disease	15,358 (16.6)	10,877 (15.4)	4481 (20.4)	< 0.001
Liver disease	1834 (2.0)	1511 (2.1)	323 (1.5)	< 0.001
Chronic kidney disease	14,952 (16.2)	11,784 (16.7)	3168 (14.4)	< 0.001
Bleeding	453 (0.5)	395 (0.6)	58 (0.3)	< 0.001
Life threatening arrhythmia	1823 (2.0)	1397 (2.0)	426 (1.9)	0.73
Shock	1272 (1.4)	943 (1.3)	329 (1.5)	0.069
Alcohol drinker	200 (0.2)	159 (0.2)	41 (0.2)	0.28
**Treatments during hospitalization, n (%)**				
Oral anti-platelet agents	35,857 (38.7)	25,312 (35.8)	10,545 (48.0)	< 0.001
Oral NSAIDs	2763 (3.0)	2276 (3.2)	487 (2.2)	< 0.001
IV diuretics	85,777 (92.7)	65,183 (92.3)	20,594 (93.8)	< 0.001
IV inotropes	10,904 (11.8)	7489 (10.6)	3415 (15.6)	< 0.001
IV vasopressors	8548 (9.2)	6220 (8.8)	2328 (10.6)	< 0.001
Respiratory support	16,430 (17.8)	10,452 (14.8)	5978 (27.2)	< 0.001
**Risk scores for stroke and bleeding**				
CHA_2_DS_2_VASc score, points	4.2 ± 1.1	4.2 ± 1.1	4.2 ± 1.2	
CHA_2_DS_2_VASc score, n (%)				< 0.001
1 point	1124 (1.2)	820 (1.2)	304 (1.4)	
2 point	4725 (5.1)	3436 (4.9)	1289 (5.9)	
3 point	15,616 (16.9)	11,937 (16.9)	3679 (16.8)	
4 point	32,172 (34.8)	24.936 (35.3)	7236 (33.0)	
5 point	28,432 (30.7)	21,743 (30.8)	6689 (30.5)	
6 point	9091 (9.8)	6729 (9.5)	2362 (10.8)	
7 point	1285 (1.4)	935 (1.3)	350 (1.6)	
8 point	121 (0.1)	80 (0.1)	41 (0.2)	
9 point	7 (0.0)	5 (0.0)	2 (0.0)	
m-HASBLED score, points	2.1 ± 0.9	2.0 ± 0.9	2.1 ± 0.9	
m-HASBLED score, n (%)				< 0.001
0 point	1880 (2.0)	1413 (2.0)	467 (2.1)	
1 point	22,614 (24.4)	18,049 (25.6)	4565 (20.8)	
2 point	39,772 (43.0)	30,426 (43.1)	9346 (42.6)	
3 point	24,436 (26.4)	17,877 (25.3)	6559 (29.9)	
4 point	3780 (4.1)	2787 (4.0)	993 (4.5)	
5 point	89 (0.1)	67 (0.1)	22 (0.1)	
6 point	2 (0.0)	2 (0.0)	0 (0.0)	

Continuous variables are presented as mean ± standard deviation. Categorical variables are presented as number of patients (%). IV: intravenous, m-HASBLED score: modified-HASBLED score, NSAIDs: non-steroidal anti-inflammatory drug, NYHA: New York Heart Association.

### Impact of heparin administration on the clinical outcomes

**Figure 2:**
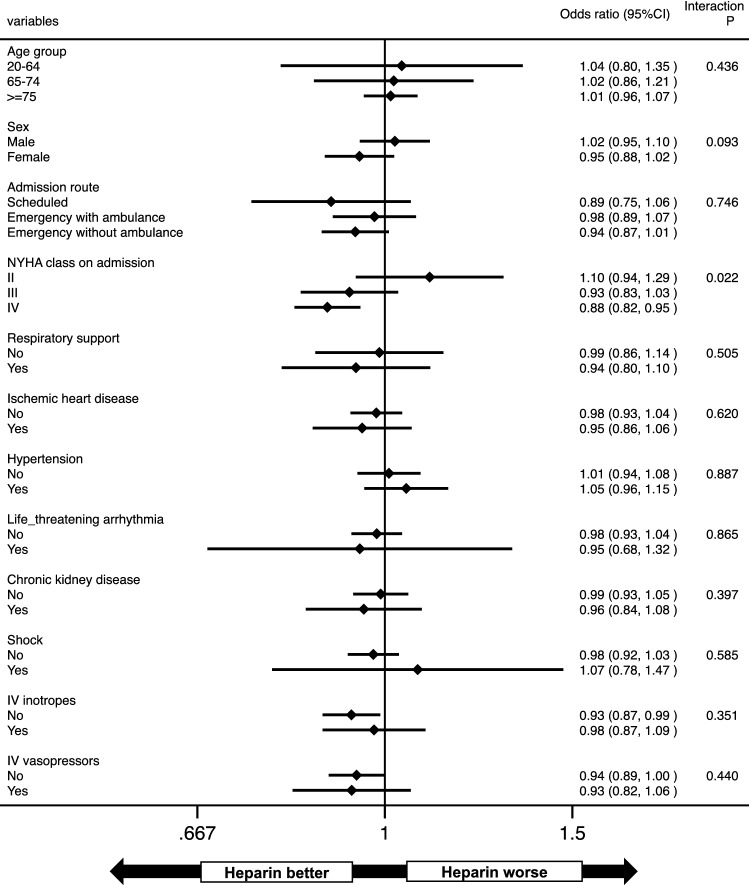
Subgroup analysis for in-hospital mortality. *CI* confidence interval, *IV* intravenous, *NYHA* New York Heart Association.

In-hospital death occurred in 9880 (10.7%) (non-heparin group 7,607 [10.8%] vs. heparin group 2273 [10.4%]) patients. Ischemic stroke occurred in 998 (1.1%) (non-heparin group 691 [1.0%] vs. heparin group 307 [1.4%]), VTE occurred in 187 (0.2%) (non-heparin group 128 [0.2%] vs. heparin group 59 [0.3%]), ICH occurred in 137 (0.2%) (non-heparin group 103 [0.2%] vs. heparin group 34 [0.2%]) and composite bleeding occurred in 963 (1.0%) (non-heparin group 759 [1.1%] vs. heparin group 204 [0.9%]) patients during hospitalization for AHF, respectively. Multivariable logistic regression analysis demonstrated that the use of heparin was not associated with the in-hospital mortality (Table 2). In subgroup analysis, a better effect of heparin on the adjusted incidence of in-hospital mortality was indicated in patients with severe NYHA class on admission (Fig. 2). However, a multivariable logistic regression analysis among the subgroups stratified by NYHA class demonstrated that the use of heparin was not significantly associated with in-hospital mortality in all NYHA groups (Supplementary Table 1).

**Table 2:** Multivariable logistic regression analyses regarding the associations between intravenous heparin therapy and in-hospital outcomes.

Outcome	Model 1		Model 2	
	OR (95% CI)	*p*-value	OR (95% CI)	*p*-value
**Primary**				
In-hospital death*	0.97 (0.91–1.03)	0.28	−	−
**Secondary**				
Ischemic stroke^†^	1.49 (1.29–1.72)	< 0.001	1.37 (1.18–1.59)	< 0.001
VTE^‡^	1.62 (1.14–2.30)	0.007	1.64 (1.14–2.34)	0.007
ICH^§^	1.18 (0.78–1.77)	0.43	1.07 (0.71–1.61)	0.75
Composite bleeding^||^	0.93 (0.79–1.09)	0.38	0.92 (0.78–1.08)	0.31

CI: confidence interval, ICH: intracranial hemorrhage, OR: odds ratio, VTE: venous thromboembolism.

*In-hospital death was adjusted for gender, age, admission route, New York Heart Association functional classification, history of hypertension, history of chronic kidney disease, history of life-threatening arrhythmia, shock, use of respirator, use of intravenous inotropes and use of intravenous vasopressor.

^†^ Model 1 was adjusted for history of hypertension, age, history of diabetes mellitus, history of stroke, vascular disease and gender. Model 2 was adjusted for model 1 with the confounders of in-hospital death.

^‡^ Model 1 was adjusted for history of hypertension, history of diabetes mellitus, history of hyperlipidemia and body mass index. Model 2 was adjusted for model 1 with the confounders of in-hospital death.

^§^ Model 1 was adjusted for history of hypertension, history of chronic kidney disease, history of liver disease, history of stroke, history of bleeding, age, use of anti-platelet agents or non-steroidal anti-inflammatory drugs and history of alcoholism. Model 2 was adjusted for model 1 with the confounders of in-hospital death.

^||^ Composite bleeding was defined as ICH and gastrointestinal bleeding during the indexed hospitalization. Model 1 was adjusted for history of hypertension, history of chronic kidney disease, history of liver disease, history of stroke, history of bleeding, age, use of anti-platelet agents or non-steroidal anti-inflammatory drugs and history of alcoholism. Model 2 was adjusted for model 1 with the confounders of in-hospital death.

**Figure 3:**
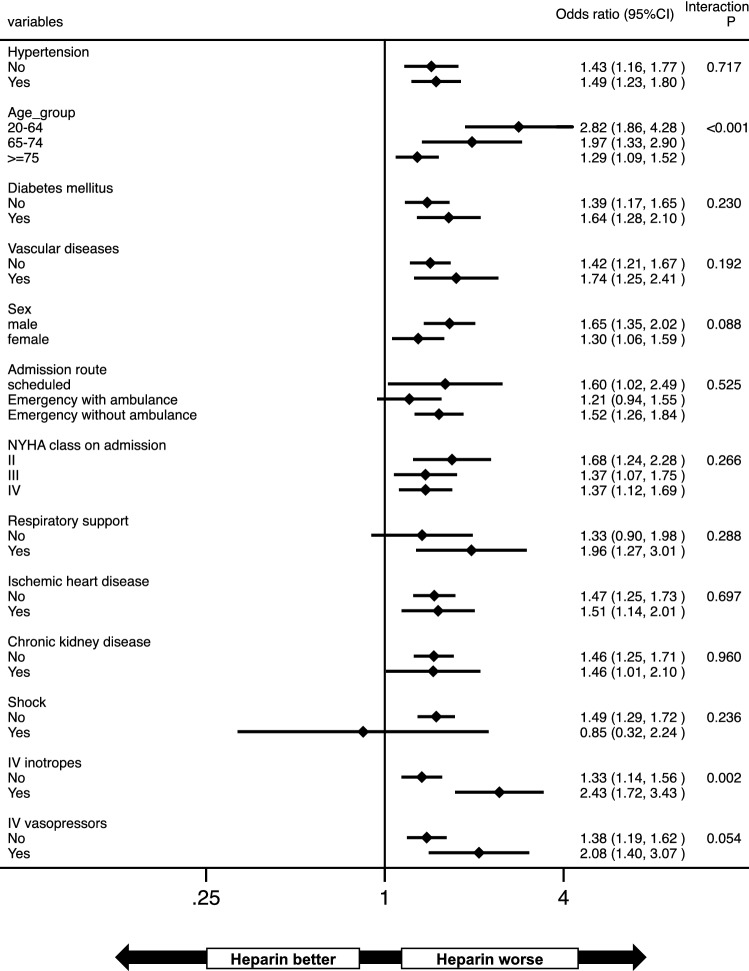
Subgroup analysis for ischemic stroke during hospitalization. *CI* confidence interval, *IV* intravenous, *NYHA* New York Heart Association.

**Figure 4:**
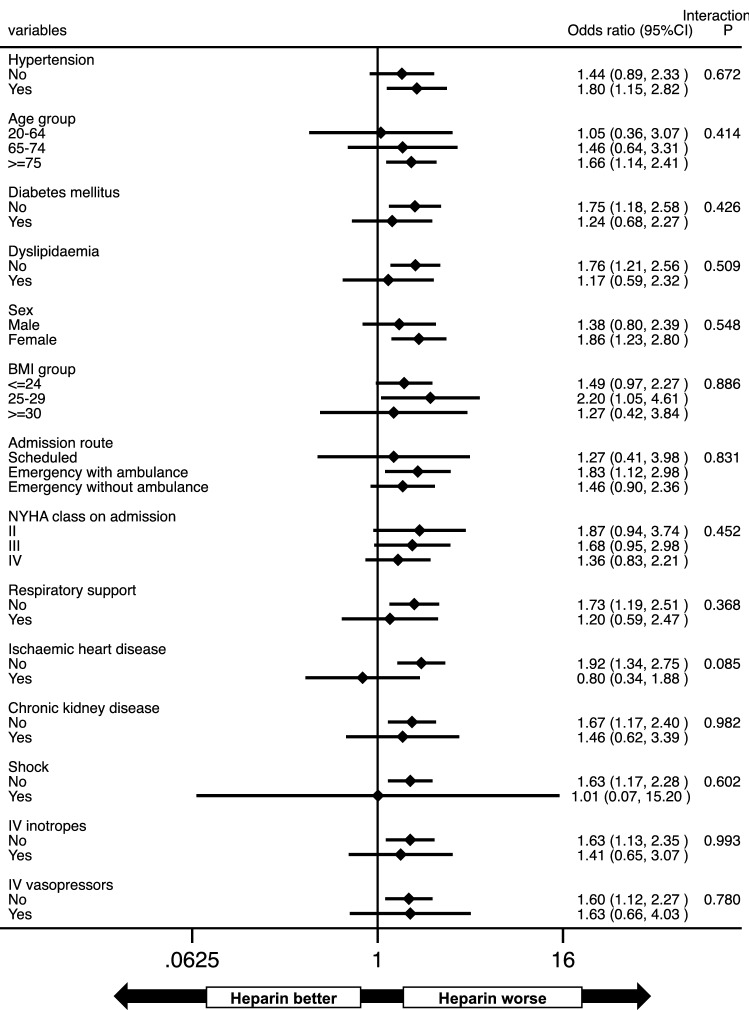
Subgroup analysis for venous thromboembolism during hospitalization. *BMI* body mass index, *CI* confidence interval, *IV* intravenous, *NYHA* New York Heart Association.

Heparin administration was not associated with the increased risk of ICH and of composite bleeding even after adjustment for the various confounders. Meanwhile, heparin use was significantly associated with the higher incidence of ischemic stroke and of VTE both in model 1 and model 2 (Table 2). In subgroup analyses, a worse effect of heparin on the adjusted incidence of ischemic stroke was observed in most patients, especially in those who were younger and were prescribed inotropic therapy (Fig. 3). A multivariable logistic regression analysis stratified by age group also showed that the use of heparin was associated with ischemic stroke, especially in patients with younger age (Supplementary Table 2). There was no significant interaction between each variable regarding the incidence of VTE (Fig. 4).

## Discussion

In the present study, we demonstrated that intravenous heparin was administered to only 24% of Japanese AHF patients with sinus rhythm despite the recommendation in the current HF guidelines^12,18^. This low administration rate was consistent with previous reports^19,20^. Furthermore, we could not find a significant association between the additive use of intravenous heparin to initial treatment and decreased incidence of in-hospital death, major bleeding, and thromboembolic events in hospitalized AHF patients with sinus rhythm.

Previous studies have demonstrated that HF is an independent risk factor for thromboembolism irrespective of AF. Mechanistically, HF augments the risk of thromboembolism via Virchow’s triad for thrombogenesis; namely, blood stasis, endothelial dysfunction and hypercoagulability^6^. Importantly, patients with AHF are considered to be exposed to higher thromboembolic risk than those with chronic HF based on the following explanations. First, high intracardiac pressure, exacerbated venous congestion, dilated cardiac chambers and reduced ventricular contraction in AHF patients can cause further blood stasis^21^. Second, a previous experimental study revealed severe impairment of endothelial function in hospitalized AHF patients^22^. Third, several studies demonstrated that patients with AHF exhibit increased hypercoagulability compared to those with chronic HF^23^. Moreover, diuretic use in the acute phase during HF hospitalization could cause hemoconcentration, resulting in an elevated risk of thromboembolism in AHF patients^9^. These exacerbations of all components of Virchow’s triad predispose AHF patients to higher thromboembolic risk.

Indeed, the incidence of thromboembolism was markedly high in the short-term period after an AHF event. We previously reported that the risk of ischemic stroke during hospitalization for AHF was elevated around 17-fold compared to after discharge^8^. Cohorts from various countries also demonstrated an extremely increased risk of ischemic stroke within 30 days after the diagnosis of HF (about 5- to 17-fold) compared with the general population^7,11,24,25^. Interestingly, a Japanese AF cohort also revealed that the risk of thromboembolism was markedly increased within the 30-day period following hospitalization for HF (hazard ratio: 12.0)^10^. These results suggest that the risk of thromboembolism peaks during the acute decompensated phase of hospitalized HF among the HF status.

Considering the risk of thromboembolism in HF patients, several randomized controlled trials investigated the efficacy of anticoagulant therapy for the prevention of thromboembolism in HF patients without AF. In these trials, warfarin therapy failed to show efficacy to reduce the composite of mortality and thromboembolism in chronic HF patients with sinus rhythm^3,26^. Recently, the COMMANDER-HF trial, which investigated the efficacy of direct oral anticoagulant therapy in HF patients with reduced ejection fraction, coronary artery disease and sinus rhythm, also could not demonstrate its efficacy on the incidence of death and thromboembolism^27^.

Notably, these studies addressed patients with chronic HF. Meanwhile, thromboembolic risk could be markedly increased in hospitalized AHF patients, as mentioned above. Therefore, the therapeutic role of anticoagulant administration, especially in AHF patients, should be further investigated. Nevertheless, there are few prospective trials regarding anticoagulant therapy in AHF patients. Previous retrospective studies showed that subcutaneous heparin therapy was not associated with reduced in-hospital mortality and 30-day post-discharge death and thromboembolic events^19,20^. In our prospective nationwide cohort study, the additive use of intravenous heparin to initial therapy was not associated with lower incidence of in-hospital death and thromboembolism in patients with AHF.

In particular, our study suggested that intravenous heparin therapy was associated with increased risk of ischemic stroke and VTE, which was contrary to our expectation. Although speculative, there are some possible explanations for this finding. First, in patients receiving warfarin, a rebound phenomenon has been reported in which the coagulation system is activated after the discontinuation of anticoagulant therapy^28^, and it is possible that the same phenomenon could also occur with heparin therapy. Second, the patients who received heparin might have been severe HF cases. In fact, the patients in the heparin group in our study had a higher prevalence of NYHA class IV, use of inotropes and vasopressors and respiratory support compared to the non-heparin group. We performed multivariable logistic regression analyses using the variables not only for risk factors of stroke, but also for factors related to in-hospital mortality of AHF; however, the severity might still not have been fully adjusted. Third, the use of heparin might have caused heparin-induced thrombocytopenia and increased thrombotic events. In fact, even after heparin is discontinued, the risk of thrombosis is 30 times that in the control population, and continues for days to weeks^29^.

The current HF guidelines recommend thromboembolism prophylaxis using heparin or other anticoagulant in hospitalized AHF patients irrespective of AF^12,18^. However, this recommendation is not based on robust evidence and/or high-quality studies, and anticoagulant therapy might be used in a small proportion of AHF patients in daily clinical practice, as was reflected in our study (only 24%). Moreover, previous reports and our study raise questions about the use of heparin for thromboembolism prophylaxis in AHF patients, indicating that routine additive use of heparin therapy to initial treatment might not be recommended in AHF patients. Considering the extremely high incidence of thromboembolism in AHF patients, we believe that risk stratification is warranted for the management of anticoagulant therapy. For instance, biomarkers such as B-type natriuretic peptide and D-dimer levels might be useful to identify high thromboembolic risk in AHF patients who are suitable for anticoagulation^8,30^. Therefore, further well-designed clinical trials following stricter risk stratification are strongly warranted to investigate the utility of anticoagulant therapy in AHF patients with sinus rhythm.

There are several potential limitations of the present study which should be acknowledged. First, despite our DPC database being confirmed by physicians, some data were based on medical claims. Therefore, these data might have had certain errors and some data might have been underestimated. The prevalence of heparin use should be interpreted with caution. Second, the JROAD-DPC database focused on JCS board-certified hospitals. We believe that this database represents current Japanese cardiovascular practice; however, there was unavoidable selection bias due to the absence of data from non-certified facilities. Third, because this database did not contain any information on blood test and echocardiogram parameters, we could have missed several major confounders for adjustment. Although the outcomes were adjusted by the risk models previously validated in the Japanese DPC database and validated risk models for each outcome, this might not have been enough to adjust the patients’ background between the groups. Fourth, we excluded invasive cardiovascular procedures where heparin was used as much as possible. Nevertheless, we might have been unable to exclude some heparin use for purposes other than prophylaxis of thromboembolism. Fifth, the length of hospitalization for AHF in Japan tends to be longer than that in other countries. Therefore, the generalizability of our results might be limited. Finally, we could not obtain data regarding the duration of heparin use and whether heparin therapy was within the therapeutic range.

## Conclusion

In hospitalized AHF patients with sinus rhythm, the additive use of intravenous heparin was not associated with lower incidence of in-hospital death and of thromboembolism, indicating that routine initial use of intravenous heparin might not be recommended in AHF patients.

## Supplementary Information

Supplementary Tables.

Supplementary Information.
