# Supplementary material for: Current practice and effects of intravenous anticoagulant therapy in hospitalized acute heart failure patients with sinus rhythm
Source: Sci Rep. 2021 Jan 13;11:1202. doi: 10.1038/s41598-020-79700-5 (PMC7807069; doi:10.1038/s41598-020-79700-5)
Supplement: Supplementary file 1 — Supplementary Tables. [file 41598_2020_79700_MOESM1_ESM.docx]

| **Supplementary Table 1.** Multivariable logistic regression analyses regarding the associations between intravenous heparin therapy and in-hospital mortality in the subgroups stratified by NYHA functional class. | | | | | | |
| --- | --- | --- | --- | --- | --- | --- |
|  | NYHA II  (n = 21,820) | | NYAH III  (n = 33,223) | | NYHA IV  (n = 37,530) | |
| Outcome | OR (95% CI) | p-value | OR (95% CI) | p-value | OR (95% CI) | p-value |
| In-hospital death^*^ | 1.11 (0.94 – 1.31) | 0.21 | 0.94 (0.84 – 1.05) | 0.29 | 0.95 (0.88 – 1.03) | 0.23 |

CI: confidence interval, NYHA: New York Heart Association, OR: odds ratio.

^*^ In-hospital death was adjusted for gender, age, admission route, history of hypertension, history of chronic kidney disease, history of life-threatening arrhythmia, shock, use of respirator, use of intravenous inotropes and use of intravenous vasopressor.

| **Supplementary Table 2.** Multivariable logistic regression analyses regarding the associations between intravenous heparin therapy and in-hospital ischemic stroke in the subgroups stratified by age group. | | | | | | |
| --- | --- | --- | --- | --- | --- | --- |
|  | Age group  20 – 64 years  (n = 8,299) | | Age group  65 – 74 years  (n = 11,643) | | Age group  ≥ 75 years  (n = 72,631) | |
| Outcome | OR (95% CI) | p-value | OR (95% CI) | p-value | OR (95% CI) | p-value |
| Ischemic stroke^*^ | 2.55 (1.67 – 3.90) | <0.001 | 1.64 (1.10 – 2.44) | 0.015 | 1.21 (1.02 – 1.43) | 0.029 |

CI: confidence interval, OR: odds ratio.

^*^ Ischemic stroke was adjusted for history of hypertension, history of diabetes mellitus, history of stroke, vascular disease, gender, admission route, New York Heart Association functional classification, history of ischemic heart disease, history of chronic kidney disease, history of life-threatening arrhythmia, shock, use of respirator, use of intravenous inotropes and use of intravenous vasopressor.
