# Supplementary material for: Current practice and effects of intravenous anticoagulant therapy in hospitalized acute heart failure patients with sinus rhythm
Source: Sci Rep. 2021 Jan 13;11:1202. doi: 10.1038/s41598-020-79700-5 (PMC7807069; doi:10.1038/s41598-020-79700-5)
Supplement: Supplementary file 2 — Supplementary Information. [file 41598_2020_79700_MOESM2_ESM.pdf]

Supplementary Appendix

Variables	
**Comorbidities**	**ICD-10 codes**
Atrial fibrillation	I48
Acute coronary syndrome	I21$, I200
Stroke (Cerebral infarction or Cerebral bleeding)	I63$ or I61$
Gastrointestinal bleeding	K920, K921, K922
Ischemic heart disease	I21$, I22$, I25.2
Dyslipidemia	E78$
Diabetes mellitus	E10$, E11$, E12$, E13$, E14$
Hypertension	I10$, I11$, I12$, I13$, I14$, I15$
Venous thromboembolism	I260, I269, I801,I802,I822, I823, I828, I829
Vascular disease	I70, I71, I671, I731, I738, I739, I771, I790, I792, K551, K558, K559, Z958, Z959
Liver disease	B18, K73, K74, K700, K701, K702, K703,K704, K709, K711, K713, K714, K715, K717, K721, K729, K760, K762, K763, K764, K765, K766, K767, K768, K769, I850, I859, I864, I982, Z944
Chronic kidney disease	N18, N19, N052, N053, N054, N055, N056, N057, N250, I120, I131, N032, N033, N034, N035, N036, N037, Z490, Z491, Z492, Z940, Z992
Bleeding (Cerebral bleeding + Gastrointestinal bleeding)	I61$, K920, K921, K922
Life threatening arrhythmias	I49$
Shock	R57$
Infective endocarditis	I33$
Heart transplantation	Z941, Z943
Alcohol drinker	E52, K70, T51, E244, G312, G621, G721, I426, K860, O354, Z714, Z721
**Procedures**	**Procedure codes**
Respiratory support	J044, J0451, J0453, J0454
Renal replacement therapy	J0381, J0382, J0383
Mechanical circulatory assist devices	K6001, K6002, K6021, K6022, K6031, K6032, K6033, K604-21, K604-22, K604-23, K604-24
**Major cardiovascular procedures**	**Procedure codes**
Cardiovascular catheter interventions	K616,K546,K556_2,K570_2,K5952,K549,K620,K5481,K5611,K5612,K5613,K595_2,K5621,K574_2,K567_2,K570_3,K5951,K615_2,K616_3,K5482,K556_2,K6153,K6153,K550,K5952
Heart surgery	K539_2, K540, K542, K543, K5441, K5442, K5443, K5521, K5522, K552-21, K552-22, K5531, K5532, K553_21, K553_22, K553_23, K5533, K5541, K5542, K5543, K5551, K5552, K5553, K557, K557_2, K557_3, K557_4, K558, K559, K5621, K5622, K563, K564, K565, K566, K5671, K5672, K5673, K5681, K5682, K569, K5701, K5702, K5711, K5712, K572, K5732, K5741, K5742, K575, K5761, K5762, K5763, K5764, K578, K5791, K5792, K579_21, K579_22, K5801, K5802, K5811, K5812, K5813, K5821, K5822, K5823, K5831, K5832, K5833, K5834, K5841, K5842, K585, K5861, K5862, K5863, K587, K588, K589, K590, K5941, K5942, K5943
Vascular surgery	K5601, K5602, K5603, K5604, K5605, K5606, K5607, K5611, K5612, K5613, K5771, K5772, K592_2, K6102, K6103, K610_4, K6105, K6141, K6142, K6143,
Transcatheter valve therapy	K6145, K6146
Intervention for congenital heart disease	K5621, K5731, K574_2
Ablation	K5951, K5952
